# Colonial vs. planktonic type of growth: mathematical modeling of microbial dynamics on surfaces and in liquid, semi-liquid and solid foods

**DOI:** 10.3389/fmicb.2015.01178

**Published:** 2015-10-29

**Authors:** Panagiotis N. Skandamis, Sophie Jeanson

**Affiliations:** ^1^Laboratory of Food Quality Control and Hygiene, Department of Food Science and Human Nutrition, University of AthensAthens, Greece; ^2^Institut National de la Recherche Agronomique, UMR1253 Science and Technology of Milk and EggsRennes, France; ^3^AGROCAMPUS OUEST, UMR1253 Science and Technology of Milk and EggsRennes, France

**Keywords:** bacterial colony, stochastic, stress response, lag time, microstructure, micro-environment

## Abstract

Predictive models are mathematical expressions that describe the growth, survival, inactivation, or biochemical processes of foodborne bacteria. During processing of contaminated raw materials and food preparation, bacteria are entrapped into the food residues, potentially transferred to the equipment surfaces (abiotic or inert surfaces) or cross-contaminate other foods (biotic surfaces). Growth of bacterial cells can either occur planktonically in liquid or immobilized as colonies. Colonies are on the surface or confined in the interior (submerged colonies) of structured foods. For low initial levels of bacterial population leading to large colonies, the immobilized growth differs from planktonic growth due to physical constrains and to diffusion limitations within the structured foods. Indeed, cells in colonies experience substrate starvation and/or stresses from the accumulation of toxic metabolites such as lactic acid. Furthermore, the micro-architecture of foods also influences the rate and extent of growth. The micro-architecture is determined by (i) the non-aqueous phase with the distribution and size of oil particles and the pore size of the network when proteins or gelling agent are solidified, and by (ii) the available aqueous phase within which bacteria may swarm or swim. As a consequence, the micro-environment of bacterial cells when they grow in colonies might greatly differs from that when they grow planktonically. The broth-based data used for modeling (lag time and generation time, the growth rate, and population level) are poorly transferable to solid foods. It may lead to an over-estimation or under-estimation of the predicted population compared to the observed population in food. If the growth prediction concerns pathogen bacteria, it is a major importance for the safety of foods to improve the knowledge on immobilized growth. In this review, the different types of models are presented taking into account the stochastic behavior of single cells in the growth of a bacterial population. Finally, the recent advances in the rules controlling different modes of growth, as well as the methodological approaches for monitoring and modeling such growth are detailed.

## Introduction to predictive microbiology

Predictive food microbiology is a sub-discipline of food microbiology that uses models (i.e., mathematical equations) to describe the growth, survival, or inactivation of microbes in food systems. Mathematical models refer to a set of basic hypotheses supporting the target (bio-) processes which are to be simulated and are possibly algebraic functions and/or differential equations (Baranyi and Roberts, 1995). Therefore, with predictive microbiology, all the knowledge of microbial responses in different environmental conditions is summarized as mathematical equations. McMeekin et al. (2008), stated that “*the model is often a simplified description of relationships between observations of the system (responses) and the factors that are believed to cause the observed responses*.”

The long roadmap of predictive microbiology over the last four decades, along with the advances toward understanding and quantifying microbial responses down to single cell level, have led to the appointment of predictive modeling as one of the most promising decision-support methodologies for food safety assessment by the Food Industry and competent authorities. Predictive modeling has been greatly benefitted by the technological and scientific evolution in both collection and processing of data, through the introduction of—omics (Rantsiou et al., 2011; Brul et al., 2012), the deployment of advanced microscopy techniques (e.g., confocal laser microscopy and fluorophores fused in the genome; Locke and Elowitz, 2009; Cox et al., 2010; Bridier et al., 2015), the application of chemometrics, data mining and the emergence of advanced data modeling techniques (e.g., artificial neural networks; Argyri et al., 2010; Panagou et al., 2011). As a next step, the rising trend for application of predictive modeling in daily practice has intensified the need to systematically exploit the vast number of available predictive models so far. Meeting this demand is being markedly achieved through the development of collective predictive modeling repositories (e.g., ComBase, Pathogen Modeling Program, iRisk, Food Spoilage and Safety Predictor, Sym'Previus, etc.). They are equipped with search engines for guided-retrieval of the appropriate food-specific or generic models (i.e., not food-specific) associated with particular hazards and built-in fitting or simulation modules, in order to visualize and numerically express the model outputs in comprehensive and ready to use formats. Such a variety of predictive models and risk assessment/risk ranking software tools may indeed help the food producers, researchers and food safety inspectors to apply the concepts of predictive modeling in quality-by-design, identification of safe product formulations and evaluation of products compliance with safety standards and microbiological criteria. A comprehensive and quite extensive review of the available software tools can be found in the study by Tenenhaus-Aziza and Ellouze (2014).

Models alone should be applied in caution and with proper disclaimers in the context of decision-making during Hazard Analysis Critical Control Point (HACCP) plan development. Use of models requires experience and judgment, both in modeling and food microbiology. Therefore, it is of vital importance to clearly perceive that the predictive models and associated software tools should not replace the expert opinion, but rather assist the experts (and sometimes even the non-experts) to elicit a food safety plan (Tenenhaus-Aziza and Ellouze, 2014). When models alone are used to make a decision, those models must be shown to be valid for the food in question and should take into consideration lot-to-lot variation. Validation may be based on published or unpublished data for very similar or identical foods. Nonetheless, even in cases when the available predictions are obtained from lab-media based models, which may potentially overlook some significant food-specific impacts on microbial behavior, such predictions are still very useful in guiding more focused and targeted challenge testing (Baranyi and Roberts, 1995; McDonald and Sun, 1999; McMeekin et al., 2008).

## Model types and classification

Based on microbial responses, expressed as change in numbers and stress tolerance, the combinations of intrinsic and extrinsic environmental determinants to which microorganisms may be exposed, are divided into the following major domains: the growth era and the domain including the combinations that allow survival or cause death of microorganisms (Booth, 2002). The conditions that lie between these two domains refer to a zone where microbial responses are uncertain and characterized by the growth/no growth interface (Le Marc et al., 2005). This zone is strongly associated with the so-called cardinal values (*T*, pH, a_w_, etc.) for growth and outlines the bio-kinetic range of microbial proliferation. Such values are species- or even strain-dependent and thus, introduce significant variability in the assessment of the impact of marginal growth conditions on microbial growth, an issue commonly encountered in quantitative microbial risk assessment. To remedy that, models have been proposed which embed the theoretical growth-limiting values for critical hurdles, such as temperature, a_w_, pH, CO_2_ and preservatives as biological meaningful parameters in the model structure. Notably, a theoretical interface also exists between survival and inactivation separating combinations that cause growth cessation but not cellular death from those that are lethal (McKellar et al., 2002).

Depending on the conceptual modeling approach applied to the target biochemical process and the final algebraic form, the models can be characterized as empirical or phenomenological, which mathematically describe specific behavior, and mechanistic or theoretical models with a biological basis, which search for the underlying mechanisms driving already observed phenomena. Polynomial equations are the most common empirical models. These models are easy to use, straightforward and no knowledge of a particular process is required. However, polynomial models have no theoretical foundation and have numerous parameters without biological meaning. Therefore, they do not offer any knowledge to mechanisms underlying a process. Polynomial models are commonly represented as quadratic response surfaces describing the environment dependence of a parameter of a bacterial population (Gibson et al., 1988).

Based on the type of dependent variable that is predicted, the models can be classified as kinetic or probabilistic. Kinetic models predict the extent and rate of growth or inactivation of a microorganism. The growth rate of a microorganism can be modeled in order to be used for making predictions based on the exponential growth of the corresponding microbial population. Kinetic models can be used to predict changes in microbial numbers with time, even if a controlling variable, which can affect growth, is changing (McDonald and Sun, 1999). This type of models constitutes a fundamental model category in predictive microbiology, especially for ready-to-eat foods, since they may assess the exposure of consumers to the doses (levels) of pathogenic bacteria at the time of consumption. The purpose of kinetic models is to estimate the time required for a specified growth or inactivation response to occur under certain intrinsic or extrinsic conditions. Such conditions include temperature, pH, a_w_, packaging atmosphere (e.g., CO_2_ levels), redox potential (Eh), the rheological properties of the food (structure-associated variables), relative humidity, nutrient content and the concentration of antimicrobial compounds (Theys et al., 2009b; Mejlholm et al., 2010; Møller et al., 2013). Thermal inactivation was the first microbial inactivation process modeled since 1920 by the canned food industry, in order to control the risk of *Clostridium botulinum* toxigenesis. First-order inactivation models were used to describe a log-linear trend of *C. botulinum* spores in low acid canned food. Through the slope of inactivation curves the thermal death time was estimated and particularly in low acid canned foods, a 12-decimal reduction (12D) of *C. botulinum* spores was shown to require exposure to 121°C/15 psi for 15 min. Over the last decades the microbial inactivation modeling was expanded to account for non-thermal inactivation (Buchanan et al., 1997). In addition to the classical linear inactivation curve, the concept of biphasic death, associated with the pre-existence or emergence of a resistant sub-populations throughout exposure to lethal conditions was modeled with non-linear models (Whiting, 1993; Geeraerd et al., 2005). Probabilistic models constitute the corner stone of predicting microbial dynamics, acting as the filter, i.e., likelihood-based decision of the primary microbial response (growth or inactivation) and guiding the selection of the subsequent kinetic modeling tool, i.e., growth or inactivation model, for predicting the change in microbial numbers in time. As such, the fate of microbial populations in foods is eventually dependent on the probability of growth or inactivation phenomena defined by the intrinsic and extrinsic factors of foods and processing environment. From a closer perspective, the behavior of an isogenic (homogeneous) population is the cumulative and stochastic outcome of its individual cells (microscopic level; Kutalik et al., 2005). Each cell within a microbial population is characterized by a variable probability for growth initiation (Koutsoumanis, 2008), followed by a stochastically defined lag time, i.e., sampled from a probability distribution (Francois et al., 2005, 2006a, 2007; Guillier et al., 2005, 2006), both resulting in a fractional growth of the total population with various sub-populations (mesoscopic level) initiating growth on different times (McKellar and Knight, 2000; McKellar, 2001). It has been suggested that under given conditions the geometric lag, i.e., the intersection of the slope at exponential phase with horizontal asymptote at the initial population level, is essentially dependent on the cumulative behavior of the fraction(s) of the initial population, which either possesses the shortest lag time (i.e., the earliest growth starters), and/or the fastest generation time (McKellar and Knight, 2000; Koutsoumanis, 2008). As a mirror image, under lethal conditions, e.g., pH < 3.0, or *T* > 60°C, the inactivation curve of a microbial population, represented by a curve of survivors (%) vs. time, is the result of the cumulative distribution of the individual cell death time, i.e., the time required to kill every single cell (Aspridou and Koutsoumanis, 2014). In explicit terms, probability models can be used to predict the likelihood of the occurrence of a microbial response as a function of intrinsic and extrinsic factors of foods and processing environment (Ross and Dalgaard, 2004). Microbial responses which have been modeled with this approach include spore germination, toxin formation by *C. botulinum*, growth initiation and survival or death of bacteria as a result of lethal pH and organic acid combinations. In the context of industrial practice, such models together with cardinal growth models may be of great assistance to HACCP, by offering science-based numerical evidence for setting critical limits, establishing process or product criteria and assessing the compliance of a given process to these limits or the legislative microbiological criteria (e.g., EC Regulation 2073/2005).

All the above model types may be further divided into the following categories, based on the combination of dependent (predicted) and independent (explanatory) variable (Whiting and Buchanan, 1993; McDonald and Sun, 1999):

- the primary models, which are used to describe the changes of the microbial population density as a function of time using a limited number of kinetic parameters (e.g., lag time, growth or inactivation rate and maximum population density);
- the secondary models expressing the effect of environmental variables (e.g., temperature, NaCl, pH, etc.) on the kinetic parameters estimated by the primary models;
- the tertiary models, which are computer tools that integrate the primary and secondary models into user-friendly units. The wider use of models in the food industry and research depends on the availability of user-friendly software (Psomas et al., 2011; http://www.aua.gr/psomas/gropin/), which encompass predictive models and allow different users to retrieve information from them in a rapid and convenient way (McMeekin et al., 2008, 2013).

The impact on microbial growth of the aforementioned intrinsic and extrinsic variables described by the models is strongly dependent on the structure of food or the model substrate. Based on that, in the following lines, a review is performed of existing modeling approaches accounting for different forms of microbial growth on surfaces, or in the interior of food matrices, either in suspension or immobilized in colonies.

### Growth rate of microorganisms in different forms of growth

In foods, microbial growth occurs in the aqueous phase. The structural characteristics (e.g., viscosity, 3D structured grid, also called “micro-architecture”) of this phase, resulting from hydrophilic structure-inducing agents, in combination with the total concentration and dispersion of water compared to fat phase determine the form and rate of growth, i.e., the spatio-temporal microbial dynamics. Food may be characterized as liquid (e.g., juices), gelled (e.g., jellies, cottage, marmelades), oil-in-water emulsions (e.g., mayonnaise, milk), or water-in-oil emulsions (e.g., butter and margarine) and the composite form of gelled emulsions (i.e., an immobilized oil-in-water emulsion). The type of emulsion determines the distribution of available water (Møller et al., 2013). The growth rate of microorganisms in response to food structure, for a given set of intrinsic and extrinsic parameters, is dependent on the motility of cells in the aqueous phase, the extent of resulting (micro-)colony immobilization and the diffusion kinetics of nutrient, oxygen, and metabolites. Three different status of growth may occur depending on the food structure:

1. If bacteria are suspended in liquids, their growth is planktonic and the motility of microorganisms may enable taxis to certain nutrient-rich sites of the food (Wilson et al., 2002). Access of cells to nutrients and transfer of metabolites away from cells contribute to the formation of a temporarily uniform environment, until the resources are depleted, or the microbial metabolites are accumulated at self-toxic levels.
2. If bacteria are growing in structured aqueous phase, e.g., due to addition of thickeners, or gelling (structure-inducing) agents, such as gelatin, pectins, starch, gums, etc., microbial cells are immobilized within the gelled regions and constrained to grow as submerged colonies in three dimensions. Their growth rates as colonies tend to be lower than that of planktonically growing cells (Wilson et al., 2002; Theys et al., 2008; Boons et al., 2013a,b, 2014; Aspridou et al., 2014). This can be further enhanced by increasing the fat concentration on the expense of water phase, thereby increasing the size of oil droplets with concomitant trend of reversal of oil-in-water emulsion. At low fat concentrations and in the absence of any dense (3D structured) network, e.g., such as that formed by proteins, the water phase may allow cell motility that resembles planktonic growth. As the fat concentration increases and compresses water, growth is constrained and becomes colonial. In a homogeneous protein network, cocci bacterial colonies are spherical while when if fat is added within a protein network, such as cheese, cocci bacterial colonies display an irregular shape. A similar effect can be obtained by adding a structure-inducing agent, such as gelatin, instead of proteins. The type and density of the gelling agent impact the growth rate of bacteria by influencing the diffusion of nutrients and metabolites to and from the colonies, respectively, as well as through the interaction of the gelling agent with inhibitory compounds, i.e., quenching or reducing diffusivity (due to entrapment) of antimicrobial agents, or due to bound of NaCl to the gelling agent (Boons et al., 2013b; Tack et al., 2015). The size and maximum achievable population of viable cells in colonies immobilized inside a structured matrix are affected by the proximity of colonies as well as the oxygen diffusion, with growth rate reduced in hypoxic or anoxic microenvironments (Noriega et al., 2008). The spatial distribution of cells of a population determines a critical population density level *per* colony, that renders the limitations in nutrient diffusion and the inhibition by metabolites sensible to adjacent colonies, leading to growth cessation (Malakar et al., 2003). Conversely, at population densities lower than the growth-limiting level, colony-to-colony interactions are negligible and cells divide without constraints. The latter critical level highly varies with the strain and the structure-inducing agent which in turn determines the chemical diffusion properties of the growth matrix.
3. If bacteria are growing on the surface of foods, such as meat and vegetables, growth is also colonial, initially in two dimensions (mono-layer), whereas the center of colony gradually develops in the third dimension most likely upward, depending on aeration and nutrient availability. Replenishment of nutrients takes place only from the bottom or the perimeter of the colony and soon cells in the center of colony experience starvation and self-toxication. This places growth constraints to the surface colony as a whole and causes suppression of the growth rate as compared to submerged growth within the food matrix or planktonic growth. Thus, the growth rate of the aforementioned different forms of growth is known to follow the order: planktonic ≥ submerged > surface (Wilson et al., 2002; Theys et al., 2008). It needs to be noted however, that the differences in growth rates between these three modes of growth likely range from significant (i.e., 0.5-or 1-fold difference) to non-significant and/or not consistent, depending on the microorganism and the structure-inducing agent (Smet et al., 2015). The same accounts for lag times. In contrast, unequivocal increase in growth rate is induced by aeration of the growth medium, e.g., in shaking culture.

These observations have also been explored in relation to the stochastic behavior of individual cells growing in liquid media or immobilized inside or on the surface of solid media (Guillier et al., 2005, 2006; Manios et al., 2012; Koutsoumanis and Lianou, 2013; Tack et al., 2015). Starting from single cell level and simulating the formation of colony or the proliferation to high numbers in planktonic state, may assist in drawing useful conclusions on the expected behavior of large populations. These aspects are further discussed in the following paragraphs.

### Individual-based modeling of planktonic or immobilized cells

The growth of a microbial population depends on the cumulative behavior of individual cells. As described above, a great variability is commonly evident in the growth responses, i.e., lag time, generation time and probability of growth, among individual cells of a homogeneous (or isogenic) population, due to the so-called “noise” (Locke and Elowitz, 2009). This biological variability (also termed “*biovariability”*; Billon et al., 1997) markedly impacts the dynamics, e.g., geometrical lag and germination time and time to reach detectable levels, of low populations such as 1–50 cells and increases with the intensity of environmental stresses (Billon et al., 1997; Smelt et al., 2002, 2008; Francois et al., 2005, 2006a; Guillier et al., 2005; Guillier and Augustin, 2006; Dupont and Augustin, 2009). For instance, the distribution of germination times (lag) of individual *C. botulinum* spores became less peaky (i.e., with lower *kyrtosis* coefficient) and less positively skewed (i.e., *skeweness* factor close to 1) as the incubation temperature decreased from 37 to 20°C, suggesting that the variance of germination times increased with the intensity of temperature stress (Billon et al., 1997; Stringer et al., 2011; Smelt et al., 2013). Variability in single cells behavior might be the result of diverse physiology of individual cells due to diverse exposure to environmental conditions in time and space of each daughter cell after the division of the mother cell. To eliminate the latter spatio-temporal diversity of the micro-environment of individual cells, microfluidic Lab-On-A-Chip systems have been proposed, such as Envirostat 2.0 (Dusny et al., 2012), which standardizes the experimental substrate under which the behavior of single cells is investigated.

However, the variable behavior of single cells is masked by the massive behavior of large populations, e.g., >500 cells or it is almost eliminated at optimal conditions (Llaudes et al., 2001; Smelt et al., 2002; Métris et al., 2006). Most of the available predictive models quantify the response of high microbial populations at a given set of conditions, which may be constant or varying with time. In order to model the variability of single cells (or single spores), stochastic modeling, i.e., where the input values are provided in the form of probability distributions describing the variability and uncertainty of the independent variables, may be applied, which is also the common approach in Quantitative Microbial Risk Assessment (Pérez-Rodríguez et al., 2007). Therefore, deterministic models, models based on input of single values for the independent variables, apply to population level, whereas stochastic models may describe the population dynamics taking into account the variability in both the input variables (i.e., extrinsic and intrinsic food parameters) and the responses (e.g., lag time and generation time) of microbial populations, either in large scale or at individual cell level.

Although deterministic models average the behavior of individual cells, the characteristics of the latter cannot be deduced from population measurements (Kutalik et al., 2005). Indeed, the growth of a population may be simulated by superimposing the evolution of independent subpopulations derived from single cells, each receiving a lag time value also termed “physiological lag,” different from the geometrical population lag (Baranyi et al., 2009), from a specific probability distribution. The evolution of a microbial population can be modeled as a Poisson birth process with constant birth intensity parameter μ (Baranyi, 1998; Baranyi and Pin, 2001). A cell capable of dividing, will divide after an initial delay consisting of the physiological lag and the generation time of the cell. Then each cell produces a subpopulation which consists of cells growing independently in the same habitat with a constant growth rate (Baranyi, 1998; McKellar, 2001). However, deviations from this rule are likely as a result of some novel non-thermal microbial inactivation treatments, such as pulsed light and electron beam irradiation, which may diversify the growth rate, due to injury, of cell clusters within a homogeneous population exposed to the treatment (Aguirre et al., 2013, 2015). For simplification purposes, to model the process of consecutive generations of cells, it is assumed that (Baranyi, 2002; Métris et al., 2003): (i) after the first division of each cell, the daughter cells enter directly in the exponential phase, suggesting that the daughter cells have no additional lag time; (ii) daughter cells do not interact by any means, e.g., competition or quorum sensing. Both assumptions were applicable when the experimental method used to describe the variability in lag times was the time to detect visible changes in the optical density of the liquid medium containing a single cell derived by a series of 2-or 10-fold dilutions of a standard concentrated microbial suspension, or even by sorting with flow cytometry (Smelt et al., 2002, 2008; Francois et al., 2003; Standaert et al., 2005; Baranyi et al., 2009). Indeed, when the population is extremely low (i.e., down to a few cells) and the volume of the liquid surrounding medium is large, it is reasonable to speculate that the interaction between floating cells is negligible or that the probability of each cell being affected by the presence and metabolic activity of adjacent cells is very low. Individual lag times commonly follow Weibull, Gamma, Exponential, or Normal distribution (Francois et al., 2005, 2006a, 2007; Kutalik et al., 2005; Métris et al., 2006; Standaert et al., 2007). The development of sophisticated image analysis systems for real-time monitoring of single cell division (or spore germination) under the microscope, during continuous exposure of attached cells to flowing liquid media, allowed further insight in the variability assessment of single cells (Billon et al., 1997; Elfwing et al., 2004). By targeting specific cells, it was observed that the generation time of daughter cells removed after division, are not the same for all cells but they follow a distribution, the variance of which, decreases with the number of consecutive divisions (Kutalik et al., 2005; Métris et al., 2005, 2006; Pin and Baranyi, 2006).

Exposure of bacterial populations to stresses (sublethal or lethal), such as chlorine, heat shock, pH, osmotic stresses, those related to minimal processing, such as irradiation and pulsed light, as well as sub-optimal conditions in a new environment shift the distribution of the time to first division to higher mean values (i.e., movement of mean to the right) and increase its variance (Francois et al., 2005, 2006a; Guillier et al., 2005; Guillier and Augustin, 2006; Dupont and Augustin, 2009; Aguirre et al., 2012, 2015). Furthermore, stress may decrease the probability of a single cell to initiate growth and increase the number of cells needed for growth initiation (Koutsoumanis, 2008; Dupont and Augustin, 2009). As a result, both extension of individual lag times and reduction of single cell growth probability may lead to false negative detection, due to insufficient growth above the threshold level of enrichment or no growth at all during enrichment (Dupont and Augustin, 2009). Given that stress increases the biological variability, interactions between cells within colonies (e.g., due to competition for nutrients, or the release of inhibitory metabolic products) may be an additional indigenous stress factor, which possibly increases cell lag variation, while retarding the growth of the total population (Guillier et al., 2006).

### Real time monitoring of single cells and derived micro-colonies

Even though the variability of growth responses (e.g., individual lag times and generation times) of planktonic cells has been extensively characterized with OD or microscopic measurements, the variability in relevant parameters of colonial growth (i.e., doubling of cells attached on biotic surfaces) associated with intra-colony cell-to-cell interactions are rarely quantified due to technical difficulties (Aguirre et al., 2012), nor even by direct imaging of cells when the daughter cell is removed after division. Thus, as an alternative, direct time-lapse imaging of microbial populations growing on agar surfaces of different intrinsic properties has enabled the characterization of population heterogeneity taking into account the interactions between adjacent cells (Koutsoumanis and Lianou, 2013) and colonies (Guillier et al., 2006). It may also depict the history of cells residing in different sites of a colony and their physiological adaptations, resulting from exposure to stresses, such as starvation or anoxia and affecting their subsequent resistance to inimical factors, e.g., sanitizers or lethal acid stress (Zhao et al., 2014; Tack et al., 2015). Experimental protocols for direct imaging of surface-growing cells include the gel-cassette system (Brocklehurst et al., 1997), the systems introduced by Billon et al. (1997), Niven et al. (2006), and later on adopted by Koutsoumanis and Lianou (2013), consisting of an agar layer on top of a microscope slide, covered by a cover slip, sealed with paraffin wax and placed under the microscope and the anopore strips (Ingham et al., 2005). Applications of these methods at single cell or colony level may be found for *E. coli* O157:H7, for which a comparison between growth rates estimated from viable count data and changes in colony area (in pixels) is made (Skandamis et al., 2007), *Bacillus cereus* in response to salinity (den Besten et al., 2007, 2010) and *Salmonella* showing the distribution of living and dead cells, during the evolution of colonies in time and space (Theys et al., 2009a). These techniques may also allow for identification of injured or even dead sub-populations within bacterial micro-colonies as a result of exposure to stresses or entrance into stationary phase of growth. This is achievable through the use of well-established fluorophores, such as SYTO 9, that stains living cells and propidium iodide that stains cells with impaired membranes (Bunthof et al., 2001). For instance, coupling direct cell imaging with viability staining, made possible to monitor the evolution of injury of various *Lactobacillus brevis* cells growing on the surface of de Man Rogosa Sharp agar, in parallel to colony growth kinetics, expressed through μ_*max*_, after short exposure to peroxy-acetic acid (PAA), i.e., an strong oxidizing agent (Zhao et al., 2014). The experimental approach applied was able to unravel some interesting physiological responses that are impossible to detect by classical plate counting. In particular, not all cells that were unable to divide after exposure to PAA were appeared red (i.e., injured), suggesting that growth inhibition was not associated with membrane damage (Zhao et al., 2014). In addition, elongation or clumps of cells experiencing starvation is a very common cellular response that denotes a highly stressful physiological state of cells that cannot be detected by plate counting and probably characterize the emergence of a viable-but-not culturable sub-population (Koutsoumanis and Lianou, 2013; Zhao et al., 2014; Tack et al., 2015).

Advances in individual-based modeling (IbM) have suggested that apart from the population measurements, the complete characterization of lag time also requires the evolution of total biomass and thus, the geometrical definition of lag time is not quite reliable (Prats et al., 2008). Furthermore, since geometrical lag depends on the time required by total viable population to exceed the detection limit of the enumeration method, a part of geometrical lag does not have practically biological meaning and can also be termed “pseudo-lag” (Koutsoumanis, 2008), because growth initiation of a fast growing sub-population, which will eventually give the detection signal, might have started quite earlier.

### Individual-based modeling in foods

The well-established variability of single cells in laboratory media is expected even more pronounced in natural food ecosystems (Ferrier et al., 2013). This may be attributable to the combination of multiple stress factors in foods, such as limitations in nutrient diffusion, competition with natural flora, accumulation of inhibitory metabolic products, structural constraints, and spatio-structural variability of microenvironments where microorganisms are located (Noriega et al., 2010). Despite the low number of studies dealing with single cell variability in foods, a common conclusion is that the behavior of low inocula (e.g., < 10 CFU/g) cannot be accurately approximated by models based on the responses of higher inocula on the same food nor by broth-based models (Schvartzman et al., 2010; Manios et al., 2012). For instance, the time that *L. monocytogenes* required for a 100-fold increase on vacuum packaged frankfurters stored at 4 and 8°C, starting from 0.007 to 0.1 CFU/g was markedly higher than that expected based on the responses of 10–20 CFU/g on the safe food (Pal et al., 2009). Likewise, the simulated variability in log-numbers of *L. monocytogenes* cells in liver pâté at 7°C or lettuce and cabbage fresh cut salads, based on broth data, differed from the observed number (Francois et al., 2006b; Manios et al., 2012). Notably, Monte Carlo simulation based on stochastic description of lag times of individual *L. monocytogenes* cells from broth data slightly over-predicted the growth of single cells of *L. monocytogenes* after 12 days on lettuce. For instance, the model predicted that there was 60% likelihood a single cell of the pathogen to reach 1.5 log CFU/g, while the observed growth under the same probability was 1 log CFU/g (Manios et al., 2012). In contrast, remarked under-estimation of the observed growth in cabbage was recorded, as the predictions showed that 60% of the individual cells could grow at 0.5 log CFU/g, whereas the observed growth was 2.6 log CFU/g (Manios et al., 2012). It is imperative that the evaluation of the response of single cells in foods should receive more focus in parallel to the optimization of laboratory media assays, which provide further theoretical aspects under controlled conditions. Furthermore, improving our ability to quantitatively characterize the micro-environment surrounding single cells, e.g., by measuring the micro-scale pH, water activity, nutrient, etc., might increase the robustness of predictions for growth or inactivation of these cells and explain their variable behavior based on spatio-temporal distribution of each cell (or cluster of cells) in the food matrix (Ferrier et al., 2013).

## Conclusion—future aspects

The deviation of broth-based predictions from the observed growth in foods is a well-known challenge of numerous predictive models, including some pioneer models of the last decades, addressing the issue of poor transferability of broth-based data to foods. Indeed, broth-based data are collected easily and under controlled conditions, thereby requiring low labor costs and assuring high reproducibility. However, such models do not adequately encompass the effect of critical factors explaining bacterial behavior in real foods, especially structured foods. Such critical factors may be associated with the constraints of colonial growth because of food micro-structure, cell-to-cell or colony-to-colony (inter-/intra-species) interactions (Habimana et al., 2011), along with the limitations due to availability of nutrients and oxygen and/or the removal of bacterial metabolites away from colonies. The impact of these factors is amplified at low microbial populations, manifested by the stochastic behavior of single cells. Thus, extrapolating broth-based predictions of microbial growth from single cells to foods may lead to significant over- or under-estimation of actual microbial behavior in foods, with important consequences on food safety and spoilage. In this context, one of the current trends in predictive microbiology is to define food micro-architecture in quantifiable (metric) variables, so that their impact on microbial growth or inactivation is quantitatively described. Advances in relevant instrumentation, such as Nuclear Magnetic Resonance (Møller et al., 2013) for assessing the distribution of water and oil particles in emulsified foods, or the development of micro-electrodes technology (Ferrier et al., 2013) measuring ion fluxes, pH or a_w_ in the food microenvironment (i.e., measurements in micro-scale; Lobete et al., 2015) have enabled the collection of useful data for the above purpose. In addition, bottom-up approaches are more and more adopted by food microbiologists in the context of predictive modeling, i.e., investigating the behavior of single cells forming adjacent micro-colonies, as a means to predict the behavior of larger microbial populations in 2 or 3 dimensions, including biofilm formation (Habimana et al., 2011). Deep insights in this area, such as stochastic description of individual lag times in the form of probability distributions, are feasible *via* the use of time-lapse microscopy (e.g., confocal laser scanning microscope), coupled with the use of viability- respiratory activity- or gene expression-associated fluorophores at single cell level (Habimana et al., 2011; Bridier et al., 2015; Lobete et al., 2015). The latter, apart from assisting in visualization of cellular division and monitoring changes in cell number in real time, they may reveal physiological trends (e.g., virulence, stress resistance, protein expression, etc.) of single cells resulting from their interaction with the liquid or solid substrate they habituate. Finally, the challenge of integrating—omics data into predictive modeling is still open and microbiologists thrive to exploit the vast amount of such type of data collected so far so as to unravel the most critical aspects of the interaction between microorganisms and foods or between cell-to-cell interactions.

### Conflict of interest statement

The authors declare that the research was conducted in the absence of any commercial or financial relationships that could be construed as a potential conflict of interest.
